# Deciphering the Role of Autophagy in Treatment of Resistance Mechanisms in Glioblastoma

**DOI:** 10.3390/ijms22031318

**Published:** 2021-01-28

**Authors:** Imran Khan, Mohammad Hassan Baig, Sadaf Mahfooz, Moniba Rahim, Busra Karacam, Elif Burce Elbasan, Ilya Ulasov, Jae-June Dong, Mustafa Aziz Hatiboglu

**Affiliations:** 1 Department of Molecular Biology, Beykoz Institute of Life Sciences and Biotechnology, Bezmialem Vakif University, Yalıköy Mahallesi, Beykoz, 34820 Istanbul, Turkey; ikhann1989@gmail.com (I.K.); sadaf.mahfooz786@gmail.com (S.M.); busrakaracam18@gmail.com (B.K.); 2 Department of Family Medicine, Gangnam Severance Hospital, Yonsei University College of Medicine, Seoul 06273, Korea; mohdhassanbaig@gmail.com; 3 Department of Biosciences, Integral University, Lucknow, Uttar Pradesh 226026, India; monibarahim@gmail.com; 4 Department of Neurosurgery, Bezmialem Vakif University Medical School, Vatan Street, Fatih, 34093 Istanbul, Turkey; burceelbasan@gmail.com; 5 Group of Experimental Biotherapy and Diagnostic, Institute for Regenerative Medicine, World-Class Research Center “Digital Biodesign and Personalized Healthcare”, Sechenov First Moscow State Medical University, 119991 Moscow, Russia; ulasov75@yahoo.com

**Keywords:** autophagy, glioblastoma, glioma stem cells, chemoresistance, radioresistance, molecular targets

## Abstract

Autophagy is a process essential for cellular energy consumption, survival, and defense mechanisms. The role of autophagy in several types of human cancers has been explicitly explained; however, the underlying molecular mechanism of autophagy in glioblastoma remains ambiguous. Autophagy is thought to be a “double-edged sword”, and its effect on tumorigenesis varies with cell type. On the other hand, autophagy may play a significant role in the resistance mechanisms against various therapies. Therefore, it is of the utmost importance to gain insight into the molecular mechanisms deriving the autophagy-mediated therapeutic resistance and designing improved treatment strategies for glioblastoma. In this review, we discuss autophagy mechanisms, specifically its pro-survival and growth-suppressing mechanisms in glioblastomas. In addition, we try to shed some light on the autophagy-mediated activation of the cellular mechanisms supporting radioresistance and chemoresistance in glioblastoma. This review also highlights autophagy’s involvement in glioma stem cell behavior, underlining its role as a potential molecular target for therapeutic interventions.

## 1. Introduction

Gliomas are the primary tumors of the central nervous system (CNS), which displays highly heterogeneous features. Among high-grade gliomas, glioblastomas are the most reported in the literature and these neoplasms are aggressive by nature [1]. Glioblastoma cells arise from abnormal star-shaped cells, known as astroglia, and develop an invasive and infiltrative nature, making its complete surgical resection complicated. Moreover, some residual brain tissue infiltrating glioblastoma cells after surgical treatment may readily cause tumor recurrence. This rapid augmentation of glioblastoma occurs due to its resistance to radiotherapy as well as chemotherapy [2]. In the recent years, a cellular recycling mechanism, autophagy, has been explained to play a crucial role in radioresistance and chemoresistance in human cancers, including glioblastoma [3,4,5].

Autophagy (self-eating) is a multifaceted and evolutionarily preserved sequence of actions that is activated in response to dysfunctional organelles and aggregated protein to sustain cellular homeostasis [6]. The autophagy pathway commences by a series of main active proteins, such as UV radiation resistance associated gene (UVRAG), autophagy-related (ATG) proteins, phosphatidylinositol 3-kinase catalytic subunit type 3 (PIK3C3), light chain 3 (LC3), Beclin 1 and ubiquitin-binding protein (p62) [7]. These proteins collectively lead to the development of double-membrane autophagosomes, and ultimately lead to the degradation of inner substances via fusion with lysosomes. Therefore, autophagy has been implicated in various diseases, including cancer [8]. Earlier, autophagy was assumed to be correlated to apoptosis [9]. Recently, a contradictory impact of autophagy, specifically the promotion of cell survival, has been widely studied [10]. This “double-edged sword” outcome (apoptosis + cell survival) in tumorigenesis varies depending upon the reaction of cells to precise stimulation and different cancer types. In the tumor microenvironment, elevated autophagy levels allow cancer cells to endure, recommencing initiation and proliferation [11]. 

Glioblastoma is a fatal disease composed of heterogeneous cancer cells of exceedingly assorted tumor microenvironments, with a poor prognosis. Despite highly developed treatment strategies, noteworthy progress in survival time remains limited. Thus, it is explained that, in the peak of the hierarchy, tumor cells or the cells capable of self-renewal and differentiation exhibit highly activated autophagy signaling to endure the specified treatment, like temozolomide (TMZ) [12]. In addition, glioblastoma pathogenesis involves the activation of survival pathways and specific genetic alterations in several genes like phosphatase and tensin homolog (PTEN), epidermal growth factor (EGFR), isocitrate dehydrogenase (IDH) and tumor protein 53 (TP53) [13]. Among these genes, EGFR is recognized as a driving receptor tyrosine kinase (RTK) in glioblastoma and induces multiple oncogenic signaling, which accounts for approximately 60% of glioblastoma cases [14]. The common mutant form of EGFR, known as EGFRvIII, is constitutively active and independent from the ligand-binding condition. In association with the EGFR signaling pathway, the melanoma differentiation-associated gene-9 (MDA-9) protein, which is related to tumor cell behavior and stemness, was amplified in glioma stem-like cells to control the defensive autophagy [15]. Furthermore, signal transducer and activator of transcription 3 (STAT3) levels were found to be related to Beclin 1 expression via EGFR amplification [16,17]. Other RTKs in glioblastoma, for instance, vascular endothelial growth factor receptor (VEGFR) [18], platelet-derived growth factor receptor (PDGFR) [19] and discoidin domain RTK 1 (DDR1) [20], also led to the alterations in autophagy via the RAF/MEK [21], Akt/mammalian target of rapamycin (AKT/mTOR) [20,22] and Hypoxia-inducible factor 1/B cell lymphoma 2 (HIF-1/BCL2) signaling pathways [23,24,25]. Consequently, therapeutic agents targeting these RTKs, such as bevacizumab and platelet-derived growth factor (PDGF) neutralizing antibody, have been known to augment autophagy signaling [18,19]. Regardless of the consequences caused by the disease/treatment-related autophagic modifications, clinical trials with autophagy inhibitors (like chloroquine (CQ)/its analogues) showed limited benefits [26]. The function of autophagy in treatment resistance in glioblastoma remains unclear, as the effect of autophagy on tumors is not universal [27]. The autophagy emergence also showed a divergent effect after different drug treatment. For instance, in a study on the inhibitor (BGT226) of the phosphoinositide-3-kinase/mammalian target of rapamycin (PI3K/mTOR) signaling pathway, the drug treatment led to cell death via autophagy in head and neck cancer cells [28]. On the contrary, in the cells sensitized with radiotherapy, the PI3K/AKT/mTOR inhibitors reduced the autophagy initiation [29]. Concerning cancer treatment, autophagy acts differently in each condition, making it complicated to understand. Conversely, the emergence of autophagy during the treatment of glioblastoma showed drug-resistance development [30]. Therefore, the present review aims to shed some light on the molecular association between autophagy, radioresistance and chemoresistance, as well as the treatment of glioblastoma. Furthermore, the potential benefits of autophagy inhibition, together with the treatment, are discussed.

## 2. Autophagy: The Process of Cellular Recycling

The word autophagy is originated from two Greek words, “auto” and “phagy”, which means self-eating [31]. Autophagy, a cell survival process, is responsible for maintaining cellular homeostasis under unusual stimuli like nutrient starvation, hypoxia, pathogen infection and oxidative stress. It is distinguished by the formation of the autophagosome, which is a double-membranous vesicle fusing with the lysosome to distribute the cytoplasmic contents for degradation. Autophagy also modulates cell health and longevity via “housekeeping” and protein quality control, which affect the regulation of ageing, immunity, neurodegeneration and cell death [32,33,34]. Additionally, autophagy increases in response to chemotherapeutic agents, thereby generating resistance and inhibiting the anticancer effect of drugs. Several examples showed that by hindering this pro-survival activity of autophagy using genetic or pharmacological agents, tumor cells could be targeted by inducing apoptosis. However, a continuous autophagic signal leads to autophagic cell death due to exhaustion of critical organelles and proteins. Therefore, autophagy plays a two-faced role in cancer, working either as a guardian or as a killer of cancer cells, depending upon the different cancer stages and the surrounding environment. Hence, targeting autophagy could be used as a promising approach for cancer treatment [35].

**Figure 1:**
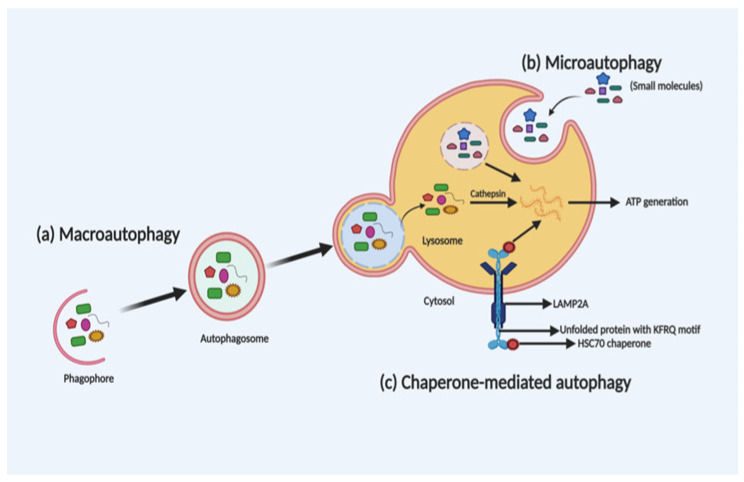
Schematic illustration of the different types of autophagy. Macroautophagy, microautophagy and chaperone-mediated autophagy (CMA) are three different types of autophagy based on the selection of the cargo and delivery approach for degradation in the lysosome. In macroautophagy (**a**), autophagosome-mediated degradation takes place by engulfment of dysfunctional organelles and aggregated proteins. In microautophagy (**b**), direct engulfment of small molecules takes place via the lysosome for degradation. Whereas, in CMA (**c**), unfolded proteins with a KFRQ motif is degraded in the lysosomes through receptor-facilitated transfer.

Depending on the selection and delivery of cargo to the lysosome, there are three different autophagy types, namely, macroautophagy, microautophagy and chaperone-mediated autophagy (CMA). Microautophagy is the only constitutive autophagy mechanism that causes engulfment of small cytoplasmic debris into the lysosome for degradation, whereas macroautophagy (the main form of autophagy) is developed in response to stress and targets the delivery and degradation of any kind of aggregated proteins and dysfunctional organelles by creating a special form of cytosolic vesicles, known as an autophagosome. CMA is also introduced in response to prolonged stress, which targets aggregated proteins with a pentapeptide motif KFRQ for degradation in the lysosome [36]. The different types of autophagy are shown in Figure 1.

Extensive research in genomics has led to the development of a new era in targeted cancer therapies that offer hope to cancer patients whose intensive chemotherapeutic treatment is improbable to give a positive result. The detection of the unique molecular characteristics in glioblastoma cells responsible for making them distinctive from the normal cell has led to the development of therapies that solely target these special kinds of genetic lesions, and overcoming chemotherapy-induced toxicity. However, the problem of resistance is found regardless of the target and the mechanism of action [37]. The presence of cell-mediated autophagy indicates a relationship between chaperones and autophagy, so it can be hypothesized that if there is any structural or functional abnormality or defect in the chaperones, it should also affect the autophagic process. Under adverse microenvironments and chemotherapy, autophagy plays a significant part in the tumor cells’ adaptation process and facilitates cell survival, which serves as a critical adaptive response during starvation and stress, leading to the recycling of energy and nutrients [38]. The dynamic role of autophagy is dependent upon the tumor development stages [39]. Autophagy may play a two-fold role in tumorigenesis by functioning equally as a tumor promoter as well as a tumor suppressor. Although autophagy may cause hindrance in cancer treatment at the late stage, it shows promise as a new target for the development of anticancer therapies for glioblastoma [40]. The influence of autophagy on tumorigenesis and treatment response gained attention after elucidation of the mechanisms behind autophagy.

## 3. Molecular Mechanism of Autophagy: Sliding on the Edges of the Sword 

Under the starvation condition, the autophagic process is divided into five main steps: (1) nucleation; (2) elongation; (3) maturation; (4) fusion; and (5) degradation. Autophagosome formation is the hallmark of the beginning of autophagy. The target molecules are encapsulated by autophagosomes, which further interacts with the lysosomes. This interaction leads to the formation of autolysosomes, where the aggregated proteins or dysfunctional organelles undergo degradation. Phagophore formation is the early stage of autophagy, which is formed by the endoplasmic reticulum, Golgi complex, plasma membrane and mitochondrial membrane [41,42,43]. The phagophore extends and sequesters the cargo proteins for degradation to form the autophagosome, which further fuse with a lysosome to generate an autolysosome. Then, the autophagosomal contents get degraded by acidic hydrolases of the lysosomes in a pH-dependent manner [44]. The formed catabolic products are implemented in several metabolic processes and undergo degradation for generating adenosine triphosphate (ATP).

**Figure 2:**
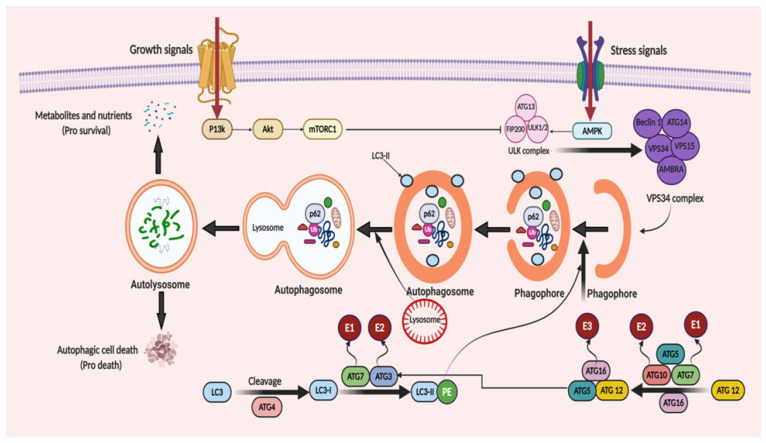
When cellular energy is sufficient, mammalian target of rapamycin complex 1 (mTORC1) becomes activated and phosphorylates each member of the ULK complex to prevent autophagy induction. In the case of glucose absence or cellular stress conditions, AMP-activated protein kinase (AMPK) becomes activated to initiate autophagic flux. This flux starts with the formation of the ULK complex consisting of ATG13, ULK1/2 and FIP200 (a ULK-interacting protein). The activity of mTORC1 is prohibited, non-phosphorylated ATG13, ULK1/2 and FIP200 forms a complex and, as a result, autophagy is started. The initiation of phagophore formation is achieved with the gathering of the ATG14, Beclin 1, VPS34, VPS15, and AMBRA proteins to generate the VPS34 complex. This also affects the further continuation of autophagic activity with downstream constituents. This takes place with the help of two ubiquitin-like conjugation systems. The first system comprises of ATG12 protein binding. Firstly, ATG7, which is linked to ubiquitin-activating enzyme E1, activates ATG12. Then, with the conjugation of ATG10 linked to ubiquitin-activating enzyme E2, ATG5 and ATG16, a huge protein complex is formed to contribute to the progression of the autophagosome. Similarly, in the second system, with the help of ATG4 protein, LC3 is modified to LC3-I. Then, LC3-I is attached to phosphatidylethanolamine (PE) to form LC3-II-PE catalyzed by ATG7 linked to E1, and ATG3 linked to E2. LC3-II-PE binds the membrane surface of the autophagosome and helps the attachment of adaptor proteins. When the phagophore formation is completed, it is named as a mature autophagosome. After fusion of the lysosome and autophagosome, an autolysosome is formed and it leads the degradation of the materials, recycles the nutrients and metabolites to supply energy (pro-survival) and triggers the autophagic cell death (pro-death).

ATGs regulate autophagy and was discovered initially in yeast [45]. Over 35 different ATGs have been identified in yeast up to date, from which some of them have their homologues expressed in mammals [46,47]. Macromolecular complexes or protein groups, for instance, the autophagy-specific PI3K complex, the unc-51 like autophagy activating kinase 1 (Ulk1) and its regulators and the transmembrane protein Atg9, regulates the initiation of phagophore formation in mammals [48,49]. The PI3K complex (active enzyme VPS34, i.e., class III PI3K, a mammalian homolog of yeast Vps15 (p150), and Beclin 1 and Atg14) catalyzes the fabrication of phosphatidylinositol-3-phosphate. This leads to the assembly of effector proteins, like the WD-repeat domain phosphoinositide-interacting (WIPI) family proteins and double FYVE containing protein 1 (DFCP1) [50,51]. Two ubiquitin-like conjugation systems (Atg12–Atg5–Atg16L and phosphatidyl-ethanolamine (PE)-light chain 3, LC3) help in the elongation process of the isolation membrane and for the formation of the autophagosome. In the first system, i.e., the Atg12–Atg5–Atg16L complex, Atg12 binds to Atg5, forming the Atg5–Atg12 complex, with the assistance of Atg7 and Atg10 (E1 and E2). The Atg5–Atg12 complex further oligomerize and develops into a larger Atg16L complex through its interaction with the Atg16L, which is localized on the outer facade of the extended autophagosomal membrane. However, the Atg16L complex gets disconnected from the autophagosomal membrane forming the autophagosome [52]. The second system, namely, the LC3 system (mammalian homolog of yeast Atg8), which is cleaved by cysteine protease, Atg4, further conjugates to the lipid PE by the action of Atg3 (E2-like enzyme) and Atg7 [53]. LC3II (a lipidated form of LC3) supplies the growing autophagosomes to further recruit cargo adaptor proteins (autophagy receptors), such as neighbor of BRCA1 gene 1 (Nbr1), NIX or p62. Adaptor proteins promote the cargo’s recruitment, such as for the damaged organelles and ubiquitinated aggregates of proteins, from the cytoplasm to the autophagosome [54,55]. Post-completion of the autophagosome formation, it fuses with lysosomes via several unknown mechanisms in a mammalian cell. A few regulators, such as LC3 (the AAA-type ATPase SKD1) and Rab7 (the small GTP-binding protein), are involved in the process of autophagosome–lysosome fusion [56,57]. Autolysosome formation then finally activates the hydrolases and cargo degradation. The molecular mechanism of autophagy is illustrated in Figure 2.

### 3.1. Autophagy Promotes Tumor Progression in Gliomas

It has been revealed that stress-mediated autophagy induction in tumor cells can lead to resistance mechanisms against the treatments with subsequent tumor progression and recurrence [4]. Noor et al. suggested that in a KRAS-driven glioma mouse model, the autophagy inhibition considerably diminished the growth of glioma and oncogenic progression by genetically suppressing ULK1 or Atg7 and Atg13, showing that autophagy is vital during the early phase in glioma pathogenesis [58]. Autophagy can also be linked to glioma progression, specifically high-grade gliomas. The levels of LC3 and p62 are notably associated with a worse prognosis, implying that LC3 and p62 could be regarded as valuable prognostic aspects of glioma [59]. Furthermore, numerous glioblastoma patients have shown an overexpression of autophagy-associated proteins with amplification of ULK1/ULK2 and transcription factor EB (TFEB) [60]. Wen et al. observed that an elevated expression of the ATG4C transcript in high-grade glioma patients was associated with shorter overall survival (OS) time. In T98G glioma cells, ATG4C knock-down repressed autophagy and this facilitated the arrest of cell cycle and apoptosis promotion via reactive oxygen species (ROS) production, p21, TP53, BCL-2-associated X protein (Bax) expressions and decreased Bcl-2 levels [61]. Reduced ATG4C expression induced the sensitivity of the T98G and U87-MG glioma cells to TMZ by autophagy inhibition. Moreover, ATG4C knock-out (KO) appreciably reduced the growth rate of glioma in nude mice [61]. The quantification of p62, LC3B, Beclin-1 and BAG3 (autophagosomal molecules) proved that nutrient or oxygen deprivation enhances the autophagy in astrocytoma compared to normal brain tissue [62]. A long, noncoding RNA (Malat1) activates autophagy and endorses cell proliferation by inhibition of miR-101, which further decreases the expression of autophagy-associated genes such as ATG4D, Stathmin 1 (STMN1) and RAB5A (Ras-related protein Rab-5A) [63]. The Malat1 levels drastically increased in glioma biopsy samples with regard to flanking normal tissue [63]. Hypoxia (~3–0.1% oxygen) stimulates the hypoxia-inducible factor 1-alpha (HIF-1α) activation, thereby promoting autophagy via regulation of transcription of autophagic genes, such as coding for the Bcl-2/E1B 19-kDa-interacting protein (BNIP3) together with the BNIP3L, ATG5 and Beclin-1 gene (BECN1) [64,65]. BNIP3/BNIP3L stimulates autophagy via Beclin-1 release from the Bcl-2/Beclin-1 or Bcl-xL/Beclin-1 complexes [66]. Besides, HIF-1α stimulates angiogenesis to ensure the oxygen and nutrient availability for tumor cell survival via VEGF transcriptional regulation [67]. The expression of angiogenic and hypoxia levels is interconnected with tumor grade and a poor prognosis in brain tumor patients [68]. Hai-Bo et al. studied that amplification in the formation of vasculogenic mimicry (VM) is associated with a poor prognosis and an elevated expression of pKDR/VEGFR-2 and ATG5 in glioma patients. Autophagy can stimulate VM through pKDR/VEGFR activation by ROS generation and the subsequent activation of the PI3K–AKT pathway in glioma stem cells [69]. These outcomes support the key role of autophagy in the resistance and aggressiveness of hypoxic glioma regions, which further supports the survival of tumor cells. These outcomes also suggest that autophagy also plays a key role in the migration, proliferation and invasion of these tumor cells. Autophagy also supports cell growth in the tumor microenvironment. Additionally, in tumor cells, oxidative stress induces the activation of pro-autophagy factors, for instance, BNIP3L, LC3, BNIP3, ATG16L, HIF-1α and nuclear factor kappa B (NF-κB) to promote caveolin-1 (Cav-1) degradation, ultimately resulting in autophagy activation. Further, extended treatment with TMZ was found to induce glioma cell line dormancy. Histone cluster 1 H2b family member K (H2BK), ephrin type-A receptor 5 (EphA5) and the insulin-like growth factor binding protein 5 (IGFBP5) have been suggested to be related to the activation of the dormancy. Interestingly, it was found to be accompanied with acquired stemness, mediated via the expression of stem cell markers, including octamer-binding transcription factor 4 (OCT4), kruppel like factor 4 (KLF4), and sex-determining region Y-box 2 (SOX2) [70]. Thus, these tumor dormancy-regulating signaling pathways could be prospective therapeutic targets to holdup or impede glioblastoma recurrence after surgery [71].

### 3.2. Autophagy Suppresses Tumor Progression in Gliomas

Autophagy has been demonstrated to inhibit the tumor initiation stage, eliminating cancer cells during tumor progression. The deletion or lower expression of important genes (Beclin-1, FIP200, blood-inducing factor 1 (Bif1), UVRAG, Atg4c and Atg5) is reported in gliomas for autophagosome initiation and elongation [72]. Earlier findings have reported the lower levels of Beclin-1 transcript in glioblastoma [73]. Notably, the elevated levels of Beclin-1 and LC3 were linked to better survival in glioma patients [74,75]. Higher AKT and mTOR hyperphosphorylation (activation) has been reported in high-grade gliomas (Grade III and IV) as compared to low-grade ones (Grade I and II) [76,77]. Additionally, the mTOR signaling pathway activation associates with the autophagy inhibition, supporting the glioma stem cell proliferation and pluripotency [78]. Likewise, glioma stem cells endorsed tumor infiltration, therapeutic resistance and malfunction of treatment [79]. MiR-224-3p suppresses ATG5 and FIP200, thereby inhibiting autophagy, and its overexpression restrained the tumorigenesis in glioblastoma cells [80]. 

The upregulation of BNIP3 (pro-cell death Bcl-2 family member) in hypoxia induces autophagy in glioma cell lines [81]. Moreover, autophagy may restrain tumorigenesis by the removal of the p62-tagged aggregates. The accumulation of p62 leads to damage of the proteins, DNA and mitochondria, as well as ROS generation, thereby promoting an unstable genome and tumor progression [82]. P62 overexpression is linked to poor prognosis in glioblastoma patients [75]. Jiang et al. showed that sinomenine hydrochloride (SE) induces autophagic cell death in glioma cells via ROS generation, consequently activating the c-Jun N-terminal kinase (JNK) pathway and inhibiting the AKT/mTOR pathway [83]. Autophagy mediates senescence; hence, it inhibits the malignant transformation as well [84]. Yuan et al. also confirmed that resveratrol improved the TMZ toxicity with increasing ROS production, which stimulates the 5′ AMP-activated protein kinase (AMPK) activation and the subsequent inhibition of the mTOR pathway, as well as decreases the Bcl-2 level [85]. Flovokawain (chalcone) inhibits cell proliferation in the U87, T98 and U251 glioma cell lines via activating autophagy and subsequent senescence mediated by ER stress; moreover, it also inactivated the AKT/mTOR pathway [86]. Autophagy may also interrupt the formation/progression of tumor through ATG protein-mediated induction of apoptosis [87,88]. It has been proposed that Beclin-1 might exert its pro-apoptotic effect with preventing the anti-apoptotic role of Bcl-xL and Bcl-2. Huang et al. suggested that Beclin-1 induced apoptosis via Bcl-xL and Bcl-2 binding, consequently releasing BCL2-antagonist/killer (Bak) and Bax, which further activates caspases-3/-9 in glioma cells [89].

### 3.3. The Bipolar Role of Autophagy

As discussed in the previous section, autophagy has been implicated in cellular defense and the oncogenic process. Although the exact mechanism of this shift is not exactly understood, it can be linked to the crosstalk between cellular apoptosis and autophagy [90,91]. Autophagy removes damaged organelles and proteins in the cell and protect the cell from their deleterious effect. In contrast, autophagy activated in response to cellular stress promotes cell survival in case of defective apoptosis. Apoptosis is a form of programmed cell death. Since autophagy and apoptosis both play crucial roles in the homeostasis of cells, the crossover of these two pathways is not surprising. These two signaling pathways cross each other at several nodes, involving interactions between (1) ATG12 and Mcl-1; (2) UVRAG and BAX; (3) Beclin1 and BCL-2; (4) Caspase and Beclin1; (5) ATG12 and ATG3; (6) ATG5 and FADD; and (7) P53 cross regulation [92,93,94,95,96,97,98,99]. These crossover nodes provide an opportunity for further investigation and also therapeutic interventions.

## 4. Autophagy Mediates Therapeutic Resistance in Glioblastoma

**Figure 3:**
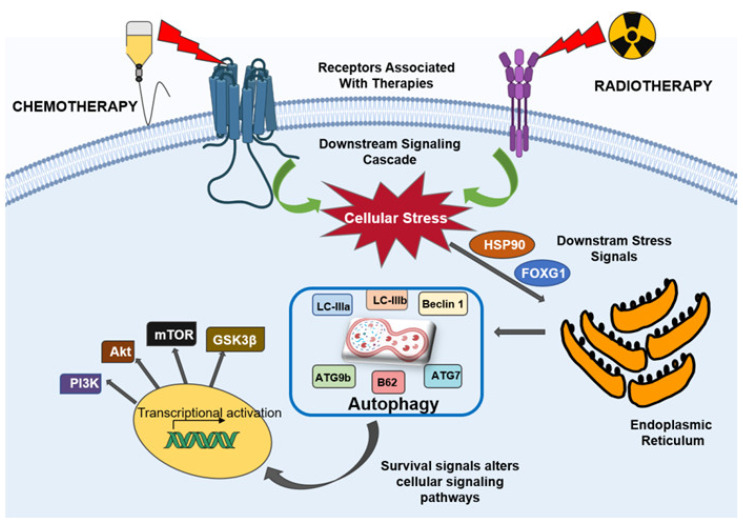
The underlying molecular mechanism of autophagy-mediated therapeutic resistance in glioblastoma.

The glioblastoma resistance to various therapies is mainly due to an extremely mutated genome and over-activation of tyrosine kinase receptors, such as EGFR, PDGFR and VEGFR, which are usually up-regulated in glioblastoma [100,101,102,103]. The ligand-mediated stimulation of PDGFR, EGFR and VEGFR activates the downstream signaling pathways, such as RAS–RAF–MAPK and PI3K–AKT–mTOR (including p38, JNK and extracellular signal-regulated kinase (ERK)), thereby transducing signals, which activate transcription factors such as NF-κB, activator protein-1 (AP-1), HIF-1α, β-catenin and forkhead box class O (FOXO). These transcription factors regulate the genes vital for inflammation, cell cycle progression, proliferation, invasion, apoptosis, autophagy and angiogenesis [104,105,106]. Owing to the altered apoptosis in gliomas, promotion of autophagic cell death could be a substitute to eliminate tumor cells. In contradiction, autophagy can mediate either cell survival or cell death in cancer cells. In cancer cells, the autophagy signaling as a death mechanism has elicited the usage of both autophagy inhibitors as well as inducers. Autophagy blockage can notably enhance the susceptibility of glioma cells to cytostatic treatments and enhance the outcome of therapies in clinical trials [107,108]. Conversely, a genetically or pharmacologically induced amplification in autophagy is associated with a significantly higher proficient tumor removal in vitro and in vivo [109,110,111]. Transcription factors, such as FOXO, p53 and TEFB, can either amplify or suppress protein expressions, thereby regulating autophagy. p53, a tumor-suppressor gene, is activated by oxidative stress or DNA damage. In the nucleus, protein expression is regulated by p53, defining the cell fate, i.e., cell-cycle arrest, apoptosis or autophagy [112]. p53 may induce the process of autophagy by enhancing autophagy induction through upregulation of liver kinase B1 (LKB1), ULK1/2 gene expression and the autophagosome. It also activates the sestrin protein expression, which activates AMPK, further phosphorylating the complex TSC 1, 2 and inhibiting mTOR, resulting in activation of autophagy [113]. Moreover, p53 enhances the suppression of regulated autophagy modulator (DRAM) expression, which strenuously stimulates apoptosis and autophagy [114]. In the case of cancer, a mutated p53 facilitates the proliferation of tumor cells and reduces the autophagy; hence, it could be a potential therapeutic target. The anti-apoptotic proteins (Bcl-xL and X-linked inhibitor of apoptosis (XIAP)) are also degraded in autophagy, consequently endorsing the elimination of tumor cells by NK cells and cytotoxic lymphocytes via the granzyme pathway [115]. Indeed, p53 activates autophagy; on the contrary, it restrains the p53 expression, promoting cancer proliferation [116]. Topoisomerase I inhibitors and chemotherapeutic agents such as cisplatin cause DNA damage and consequently activate ataxia-telangiectasia mutated (ATM) protein kinase via phosphorylation. Subsequently, ATM phosphorylates PTEN, allocating its nuclear translocation for autophagy induction by restraining the AMPK pathway [117]. Hence, autophagy has been suggested to support tumor cell endurance. The Fox O family of transcription factors is a different autophagy regulator. Four members of the Fox O family, namely Fox O1, O3, O4 and O6, have been depicted in mammals [118]. Fox O proteins are cytoplasmic and translocate to the nucleus to stimulate the expression of many genes, counting the ones that regulate the autophagy induction (ULK1, ULK2 and sestrin 3 (SESN3)), elongation (ATG4, ATG5, ATG12, GABARALI and microtubule-associated protein 1 light chain 3 beta (MAP1LC3B)) and autophagosome–lysosome fusion (TFEB and Rab7) [119]. Fox O can also modify autophagy at different levels via epigenetic mechanisms or intermingling with autophagy regulators (for instance, Atg7) [120,121]. On the contrary, autophagy can also regulate the expression of the Fox O transcription factor. Primarily, TFEB is associated with lysosome biogenesis; however, it is also involved in the modulation of autophagy activation during starvation [122]. Glycogen synthase kinase 3 beta (GSK3B), ERK2 and mTOR are the focal kinases, which phosphorylate and sequester TFEB in the cytoplasm [123,124]. Although, upon dephosphorylation, TFEB translocates to the nucleus and regulates several processes, such as the formation of autophagosomes and the fusion of autophagosomes and lysosomes. In fact, upon phosphorylation by ERK2 and GSK3B, the nuclear translocation of TFEB is inhibited and, thus, autophagy is decreased [124]. TFEB has been reported to be overexpressed in high-grade gliomas along with the extra genes coding for the autophagy proteins, such as Beclin 1, LC3A, LC3B, Ulk 1, Ulk 2, p62 [60] and sequestome 1 (SQSTM1) [125]. Interestingly, the inhibition of the WNT pathway increases the expression of SQSTM1 in glioblastoma cells and sensitizes the tumor cells to the autophagy inhibitor effects, and therefore could offer an alternative therapy [125]. The mechanism of autophagy-mediated therapeutic resistance in gliomas is depicted in Figure 3.

### 4.1. Autophagy in Chemoresistance

The ability of cancer cells to avoid cell death in the presence of chemotherapeutics is known as chemoresistance. Despite several studies, overcoming the chemoresistance remains a challenge for most cancer therapies [126]. Unlike other cancers, having the blood–brain barrier itself is a massive barrier for chemotherapies in brain tumors [127]. During the past decade, several studies have revealed different molecular mechanisms, including signaling pathways and cellular molecules that play a crucial role in driving therapeutic resistance in gliomas. In recent years, autophagy has gained attention in terms of its role in tumor initiation and chemotherapy response mechanisms. The cellular stress conditions, such as nutrient deprivation, DNA damage or hypoxia, induce enhanced autophagy. As highlighted by several recent studies, autophagy-mediated pro-survival signals drive the resistance to anti-angiogenic chemotherapies in glioblastomas [3,128]. Activated autophagy within cancer cells in response to therapeutic drugs, which consequently desensitizes the cancer cells and facilitates cell survival, is also known as protective autophagy [129,130]. 

#### 4.1.1. Cellular Pathways in Chemoresistance

High-grade gliomas show limited sensitivity towards chemotherapies, considering the role of autophagy in drug-resistance mechanisms, pharmacological inhibition or knockdown of autophagy genes, and may serve as a potent approach to overcome drug resistance in glioblastoma [131]. Wang et al. investigated the effect of glucose starvation on autophagy-mediated chemoresistance in patient-derived glioblastoma U251 and U87 cell lines. This study showed that glioma cells in deprived glucose conditions demonstrated enhanced cellular autophagy. Furthermore, they indicated that alleviated autophagy is providing resistance against chemotherapy by enhancing survival, quiescence and reprogramming cellular metabolism [132]. Treatment of U373 and U87 cells with cisplatin induced autophagy, but knockdown of retinoblastoma protein inhibited autophagy and sensitized glioblastoma cells to apoptosis. The combinations of chemotherapy with autophagy inhibitors are found to be improving the therapeutic efficacy in glioblastoma [131]. Several glioblastoma tumors demonstrate widespread endothelial proliferation accompanied by high expression of VEGF [128]. Bevacizumab is a well-known inhibitor for VEGF and has been implemented in treatment regimens for patients with gliomas. In several cases, gliomas do not respond to bevacizumab through inducing autophagy [128]. Hu et al. demonstrated hypoxia-associated autophagy in glioma xenografts when treated with bevacizumab [23]. Elevated autophagy was also accompanied with enhanced survival resistance to bevacizumab. However, co-treatment with CQ and bevacizumab, which are known autophagy inhibitors, reduced the growth of tumor. These experimental findings reveal a unique means of resistance to anti-angiogenic therapy, where cell survival is promoted by hypoxia-mediated autophagy [23]. The presence of hypoxic core regions is one of the important characteristics of glioblastoma, which are known to have an association with chemoresistance and tumor aggressiveness. A very recent study explained the role of the hypoxic state in patient-derived glioma cells to escalate the antigenic and invasive potential. The aggressive nature was accompanied by upregulated autophagy-related genes, including BCL-2 interacting protein 3 (BNIP-3) and DNA damage inducible transcript 4 (DDIT4), which are associated with chemoresistance [133]. EGFR, VEGF and RET tyrosine kinase are shown to be a contributing factor in the underlying drug-resistance mechanisms in glioblastoma patients. Further, Shen et al. suggested that implementing small molecule inhibitor ZD6474 (Vandetanib) could induce autophagy in glioma cells to overcome the cytoprotective adaptive response against chemotherapy [134]. Their findings suggested ZD6474 induced autophagy in U251 and U87 cells, mediated through the PI3K/Akt/mTOR signaling pathways. It was explained that knockdown of ATG7 and Beclin 1, which are essential autophagy genes, sensitized the glioma cells in vitro. Moreover, the U251 cells xenograft model, when treated with ZD6474 in combination with the autophagy inhibitor CQ, significantly inhibited tumor growth, thereby indicating the protective role of autophagy in glioblastoma cells [134]. In a similar study, NVP-BEZ235, an inhibitor of the PI3K/mTOR pathway, was shown to induce autophagy in glioma LN229, U87, U373 and SF763 cells in vitro and enhanced survival in glioma xenograft models [135]. Additionally, NVP-BEZ235 in combination with CQ inhibited autophagy and induced a reduction in tumor growth in vivo [135]. These findings describe the potential of inhibiting the PI3K/mTOR pathway to target autophagy-mediated chemoresistance in glioblastomas. The TGFβ/SMAD pathways also have been found to enhance the epithelial-to-mesenchymal transition (EMT), giving rise to chemoresistance in glioma cells [136]. They explained that small HECT and RCC1-like domain (HERC3) was found to be highly expressed in pseudopalisade cells around a tumor’s adjacent tissues and in tumor necrosis. It was found that HERC3 regulated autophagy in tumor cells via downregulating SMAD7 expression. In vitro and in vivo findings confirmed the direct association of HERC3 expression with poor survival and directed towards reduced sensitivity towards treatment [136]. Currently, several studies have attempted to evaluate the function of long non-coding RNAs (lncRNAs) in various cancer types. Although their functions on chemoresistance have been well documented, its role in autophagy-mediated chemoresistance in glioma cells have not been well defined. To clear this obscurity, Jiang et al. investigated the effect of cancer susceptibility 2 (CASC2) lncRNA on chemoresistance in glioma patients. Based on their findings, they reported that expressions of CASC2 and negatively regulate miR-193a-5p expression. Upregulated miR-193a-5p decreases the mTOR expression and thus consequently decreases the protective autophagy. CASC2 is downregulated in glioma cells and thus increases autophagy through the mTOR signaling pathways. These finding indicated the importance of CASC2 lncRNA as a potential therapeutic target for therapeutic intervention [137]. 

#### 4.1.2. Targeting Autophagy to Overcome Chemoresistance in Glioblastoma Cells

It is known that TMZ causes cell death in glioma cells by apoptosis induction. Interestingly, TMZ also induces autophagy that drives resistance mechanism, enhancing the survival of glioma cells [30,68,138]. TMZ is the primary chemotherapeutic implemented for the treatment of glioblastoma patients; however, a novel adjuvant therapy capable to overcome chemoresistance would be beneficial for the patients. MicroRNAs (miRs) are explained to regulate chemosensitivity in glioma cells mediated through autophagy [139]. A recent study demonstrated the role of miR-519a to overcome TMZ resistance in chemoresistant U87 cells via inducing autophagy through inhibiting the STAT3/Bcl-2 signaling pathways [140]. The study points out the therapeutic potential of miR-519a in combination with TMZ. Momelotinib (MTB), an inhibitor of Janus kinase (JAK)-1/2, induced a growth inhibitory effect on U251 glioma cells in vitro and in vivo [141]. It was shown that MTB induced autophagy in chemoresistant glioma cells via upregulating the expression of LC3-I, LC3-II, Beclin1 and p62 proteins. Moreover, the combination of MTB and TMZ reduced phosphorylation of AKT and STAT3 alongside the overexpression of the autophagy proteins [141]. With the recent updates in the plant-derived small inhibitor molecules with anticancer activities, such research has gained more attention. Liu et al. explained the cytotoxic efficacy of polyphyllin VIII (PP7) in glioma cell lines through inducing cellular autophagy by inhibiting the AKT/mTORC1 pathways [142]. Their study indicated that PP7 in combination with TMZ induced autophagy via suppressing p62 while increasing the ratio of LC3 II/ LC3 I protein expression in glioma cells, which was mediated by ROS [142]. Similarly, GANT-61 was shown to induce ROS-mediated autophagy activation in U251 cells [143]. A recent study demonstrated that combination treatment of simvastatin and TMZ induced apoptotic death in U251 cells via inhibition of autophagy [144]. This combination treatment inhibited the fusion of the lysosome to the autophagosome, which consequently resulted in the accumulation of intracellular autophagic cargos [144]. In a similar study, lovastatin was shown to impair the autophagic flux through inhibiting the AKT/mTOR pathways [145]. Although, several other studies have attempted to shed light on the underlying mechanism of chemoresistance in search of novel therapeutic targets. A very recent study explained the association of aldehyde dehydrogenase (ALDH) enzyme with chemoresistance in glioma cells [146]. Oxidative stress leads to the generation of active aldehydes as a result of lipid peroxidation, and these aldehydes are detoxified by the ALDH enzyme. It was found that TMZ-induced protective autophagy reduces ALDH expression, thereby contributing to the chemoresistance in glioma cells [146]. Similarly, Yun et al. recently explained the role of the DOC-2/DAB2 interacting protein (DAB2IP), which is a member of the Ras-GTPase activating proteins in chemoresistance in glioblastoma cell lines [147]. DAB2IP enhanced the sensitivity of A172 and U373 cells to TMZ via suppressing the protective autophagy through downregulating ATG9B expression. Moreover, their findings explained that expression of ATG9B was negatively regulated through the Wnt/β–catenin signaling pathway [147]. In this context, findings of our laboratory have shown anvirzel to downregulate the expression of glycogen synthase kinase-3 beta (GSK3β), consequently stimulating autophagy in U87 cells [148]. Collectively, small inhibitor molecules demonstrate promising efficacy in targeting autophagy to overcome chemoresistance in glioblastoma cells and should be pursued further. However, it is crucial to address the question that what degree of autophagy modulation will facilitate the sensitization of glioma cells towards chemotherapies. In an interesting study, Kriel et al. explained the extent of autophagy modulation required to sensitize and induce a cytotoxic effect in glioma cells. Pre-treatment with an autophagy inducer followed by an autophagy inhibitor and TMZ impaired the ability of glioma cells to evade drug-induced cell death. Moreover, the molecular basis was associated with a reduced mitochondrial electron transport chain and alteration in oxidative phosphorylation [149].

### 4.2. Autophagy in Radioresistance

As a normal response mechanism, autophagy can be activated in mammalian cells when treated with ionizing radiation (IR) [150]. Currently, the role of autophagy in cancer cells in response to radiotherapy is not completely understood; also in the case of malignant brain tumors, the outcome of radiation-induced autophagy is bifunctional, including both protective and cytotoxic effects [151,152]. Several studies support the fact that IR at a clinically relevant dose can induce autophagy in tumor cells [153,154,155,156]. Autophagy is thought to assist tumor cells to evade cell death through the cooperative crosstalk between the apoptotic signaling pathways [91,157]. Tumor cells irradiated with IR is endangered by cellular toxic species, including, misfolded proteins, free radical species and damaged organelles, which lead to cell death [158]. Several studies have reported the pro-cell death role of autophagy; however, autophagy can scavenge damaged organelles and proteins, which can be associated with its protective role in maintaining the proliferation of irradiated cancer cells [159]. Radiotherapy in brain tumor cells induces autophagy through a different cellular mechanism, such as PI3K/AKT/mTOR, the mammalian STE20-like protein kinase 4 (MST4 kinase)–ATG4B signaling pathways and intracellular ROS [160,161,162]. Thus, inhibiting autophagy in tumor cells makes them susceptible to radiation treatment; for instance, inhibition of MDA-9 sensitized glioblastoma cells through autophagy induction [156,163]. Glioma cells with different radio-sensitivity demonstrate a separate response. Sensitive glioma cells, when treated with a low fraction of radiotherapy, results in an increase in autophagic flux; on the contrary, relatively radioresistant cells treated with a higher fraction of radiotherapy inhibits autophagic flux [164]. It is shown that CD133+ glioma stem cells (GSC) have a low level of the activated autophagy pathway [165,166]. Moreover, treatment of GSCs with radiotherapy in combination with bafilomycin inhibited autophagy and sensitized the GSCs toward radiation treatment [167]. However, to date the exact mechanism of association between autophagy and radiation in GSCs is unknown.

The effect of radiotherapy being enhanced by targeting autophagy is still debatable. One study revealed that, after radiation, autophagy induced apoptotic cell death in malignant glioma cells, and by reducing the function of autophagy, it can be reversed [168]. Contrary to this, other studies showed that dual inhibitor (NVP-BEZ23) of the P13K/mTOR signaling pathway can enhance autophagy through affecting the ATG5 and Beclin1 autophagy-linked proteins and DNA damage repair proteins functions, and can stimulate the radiation sensitive glioblastoma cells [169,170,171]. These results revealed diverse reactions after radiation and inhibitor addition, which ultimately resulted in cell death. Another report also showed that rapamycin led to autophagy activation, which resulted in glioma stem cells differentiation, a decline in tumorigenesis and enhancement in the sensitivity of glioma cells towards radiotherapy [172]. Although, data related to the use of inducers for autophagy in clinical trials as well as on mice experiments are still limited [169,173]. Moreover, currently, in most of the clinical trials, they prefer to combine inhibitors of autophagy with radio-chemotherapy rather than with radiotherapy alone. Hypofractionated stereotactic radiotherapy (HSRT) has provided a treatment alternative to clinicians as it provides protection to the adjacent normal tissues and delivers a sufficient radiation dose to the tumor site. Findings from our laboratory have shown that HSRT downregulates the expression of heat shock protein 90 (HSP90) and inhibits proliferation of glioblastoma U87 cell proliferation dose-dependently (unpublished data). In a very recent study, Zheng et al. revealed that radioresistance-associated long intergenic noncoding RNA 1 (linc-RA1) was increased in radioresistant glioma tissues and glioma cells as compared to non-tumor tissues and radiosensitive cells. Linc-RA1 was related to further clinical situations and a lower overall survival. Linc-RA1 enhanced glioma radioresistance in vivo and in vitro. Their findings revealed that Linc-RA1 inhibited the interaction between ubiquitin-specific protease 44 (USP44) and H2B K120 monoubiquitination (H2Bub1), which consequently inhibits autophagy and promotes radioresistance in glioma cells. Thus, we can speculate on the importance of Linc-RA1 in autophagy-mediated radioresistance, as well as on its possible role as a therapeutic target or biomarker [174]. 

#### Targeting Autophagy to Overcome Radioresistance in Glioblastoma Cells

Zheng et al. created a radioresistant subclone of the U251 cell line and showed that cathepsin D (CTSD), which has a role in glioma prognosis, had increased expression levels in the radioresistant subclone U251 cell line. Silencing of the CTSD gene by its inhibitor, pepstatin A, or by small interfering RNA (siRNA), decreased the radioresistance. The autophagy level increased in the radioresistant glioblastoma subclone cells compared with the U251 cells. Silencing of autophagy by use of LC3 siRNA sensitized the glioblastoma cells to the radiation treatment. Furthermore, the CTSD protein expression level was negatively correlated with p62, a classical receptor of autophagy, and positively correlated with LC3 II/I in radioresistant cells. CTSD inhibition reduced the development of autolysosomes, while enhancing the development of autophagosomes, which showed a deteriorated autophagy level, causing radiosensitization. This study showed for the first time that CTSD modulated the radioresistance of glioblastoma by influencing the fusion of lysosomes and autophagosomes. The use of CTSD can be a powerful candidate for glioblastoma therapy [175]. 

DDR1, which is a type of cell adhesion molecule, causes radioresistance in glioblastoma stem-like and bulk cells by its adherence to the extracellular matrix and after modulation of autophagy/macroautophagy. DDR1 relates to a YWHA/14–3-3-BECN1-AKT1 multiprotein complex favoring the pro-survival/anti-autophagic and resistance-mediating AKT-MTOR pathway. Silencing of DDR1 reduces the radioresistance of glioblastoma cells via inducing autophagy. Consequently, targeting DDR1 might be a novel approach for sensitizing glioblastoma cells to radiation treatment [20]. Mitrakas et al. showed that radioresistant T98 glioblastoma cells, when treated with 4 Gy radiation and after silencing of the TFEB, LC3A, and LC3B genes, presented increased autophagic flux. However, relatively radiosensitive U87 cells sustained obstruction of autophagic flux. When the TFEB, LC3A, and LC3B genes were silenced, the U87 and T98 cell lines became more sensitized to radiation. In addition, mouse xenografts were sensitized towards radiation with silencing of the LC3A gene. These findings indicated that therapy of glioblastoma patients could be more effective with a combination of autophagy blocking and radio-chemotherapy [176]. So, this prospect’s investigation should be more focused towards the assessment of factors involved in radiation-mediated autophagy induction and the effect of this altered autophagy on the cells. Forkhead box G1 (FOXG1) was increased in glioma tissues, which plays an important role in proliferation, differentiation and regulation of the cell cycle. Xiao et al. demonstrated that the expression level of FOXG1 was increased in U87 and TG-905 glioma cell lines after radiation. The expression of FOXG1 weakened the radiosensitivity of the glioma cell lines via inducing autophagy. FOXG1 might be a target for glioma treatment and it is a significant object to overcome radiation-enhanced cell death in deadly glioma cells by the modulation of autophagy [177].

## 5. Glioma Stem Cells: The Undercover Resistance Mediators

Owing to progress in the cellular and cancer biology methods of investigation, the mechanisms of glioblastoma resistance were uncovered in the last decade, with several studies showing that glioblastoma heterogeneity influences the unequal response of cancer cells to therapy [178]. Cancer stem cells (CSCs) are a subtype of the tumor population that plays a central role in glioma progression and resistance to therapy [155]. Previously, it has been shown that CSCs can produce neurospheres in vitro, forming tumors in an immunocompromised animal model in vivo, and showed resistance to TMZ and ionizing radiation [78,179]. Therefore, understanding the molecular mechanism of CSC resistance and survival, mediated by therapy, will have a credible benefit for future anticancer approaches and possibly improve the patient’s survival.

To date, only 3–5% of patients have survived for more than 3 years [180]. Although O-6-methylguanine–DNA methyltransferase (MGMT) promote methylation and the status of the IDH1 mutation seem to show correlation with longer survival, recurrence of glioblastoma patients is almost common. Moreover, recurrent glioblastoma tumor usually behaves far more aggressively with a median survival of around 5–7 months [181,182]. Even though the molecular pathways implicated in resistance to TMZ and IR has been extensively studied, our understanding of the molecular mechanisms of glioblastoma resistance is yet to be investigated. Previously, chemoresistance to TMZ in glioblastoma cells was mainly associated with MGMT methylation status [183]. Since then, it has been noticed that many cancer cells had gained an upregulation of CSC transcriptional factors, which promotes glioma stemness, EMT, angiogenesis, etc. [184,185,186]. Overall, the expression of these transcription factors along with the expression of their downstream and upstream targets comprise a chain of events that is called an adaptive stress reaction, which is detected during chemotherapy and tumor surgical resection [187]. It has been reported earlier that CSCs heavily relies on several signaling pathways, which support their behavior and survival. Autophagy is a process that gives CSCs the ability to survive via activation of pro-survival signaling. The canonical autophagy targets and degrades organelles and proteins in the lysosomes. To perform this process, the ATG1/ULK1 complex initiates autophagosome formation, phosphorylates the PI3K-based complexes and promotes membrane recycling [188,189]. Besides, multiple pathways and genes associated with metabolic and environmental stress have been recovered, suggesting that autophagy modulation might be a new strategy to fight glioblastoma. Several autophagy inhibitors and its derivates have been extensively studied in vitro in several groups of glioblastoma patients [190,191]. Although testing them in vitro using various glioblastoma cells clearly shows the benefits for anticancer therapy, the precise mechanism of their therapy remains unclear. For instance, it is known that these drugs produce several non-autophagy-related side effects that might impact glioblastoma growth [192]. From another side, experimental drugs, oncolytic viruses and chemotherapeutic drugs, such as TMZ, require inducted autophagy, which appeared to increase their anti-glioma effect [193]. Therefore, under different circumstances, autophagy can promote and inhibit glioblastoma progression.

### Artificial Autophagy in Glioblastoma: A Tale of Intrigue

The next question is whether GSCs develop autophagy signaling over the course of the disease and what are the markers for autophagy induction in glioblastoma? Is it feasible to distinguish between the markers of prosurvival autophagy and autophagy that contributes to cell death? In this case, the data is also conflicting. An important issue to consider is the type of tissue produced by malignant cells that is available for analysis. A major problem in monitoring autophagy using patient-archived samples was based on several investigators’ opinion that autophagy induction or blockade of the autophagic flux may impact the expression of C3B and p62 [194]. To elucidate whether primary glioblastoma exhibit expression of pro-survival autophagy, Jennewein et al. analyzed 350 astrocytomas for the expression of autophagy-related markers, including p62, Beclin1, LC3B, BAG cochaperone 3 (BAG3), cathepsin B (CTSB) and lysosome-associated membrane protein 2 (LAMP2) lysosomal markers [62]. They observed no difference in the Beclin1, LC3B or p62 levels among the tumor grades that were compared with normal CNS specimens, suggesting normal cell maintenance for glioblastoma tissue. At the same time, BAG3, a non-canonical inducer of autophagy, was upregulated in reactive astrocytes, allowing to connect two distinct pathological states such as glioblastoma progression and ischemia [195]. Most recently, Guadagno et al. tried to set up the connection between Beclin 1 expression and the clinicopathological features of glioblastoma [196]. As in the first instance, no correlation was found, suggesting no difference between autophagic activity in normal brain samples and glioblastoma samples, and no activation of Beclin 1, LC3 and P62 was connected to glioblastoma progression. Although, our bioinformatical assessment of GEO-deposed glioblastoma tissue reveals the expression of mRNAs for several glioblastoma markers [196]. We believe that conducting an immune-histochemistry (IHC) analysis is a true assessment of the autophagy status, as they are in the glioblastoma tissue. Using the IHC technique, the authors can distinguish glioma tumor cells from immune cells, which can exhibit their autophagy profile, as different from fibroblasts, neural stem cells, etc., and not distinguished from the total RNA sequence. 

**Figure 4:**
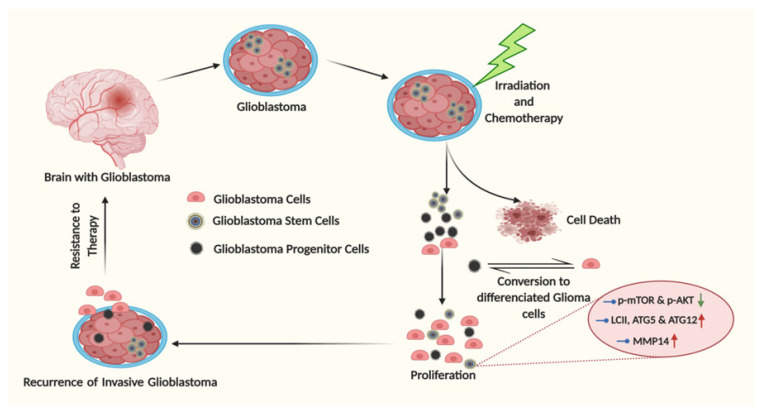
Tumor progression and recurrence in glioblastoma tumors mediated by glioma stem cells (GSCs). GSCs showed self-renewal and induced glioblastoma progenitor cells generation, which further transformed into differentiated glioblastoma cells. This differentiation causes higher proliferation and invasive glioblastoma recurrence, which leads to resistance towards different therapeutics. GSCs are capable to re-initiate the progression of tumors. Pathways, including mTOR, AKT and MMP14, and the molecules related to autophagy, including LCII, ATG5 and ATG12, get altered in GSCs. This figure illustrates the involvement of cellular flexibility in recurrence of illness and could help to develop new therapeutics to inhibit recurrence.

However, the detailed biology of glioblastoma in terms of autophagy regulation came from the in vitro cultivation of primary glioblastoma samples. It was established that CSCs, the main source of glioblastoma resistance, can be propagated in 3D cell cultures with the usage of growth factors and serum-free medium. Although, these conditions are neither ideal nor fully mimics the CSC propagation in a patient’s brain in the presence of patient serum. The spheroid cultivation system may be a good source for cell signaling characterization in tumor cell subsets. The majority of publications that are available through the Pubmed portal, in which GSCs are grown in serum-free conditions, were used to analyze the expression of the pro-autophagy isoform II of LC3 protein. GSCs exhibit a decreased level of the pro-autophagy isoform in the presence of TMZ [197]. This is correct for patient-derived GSCs and adherent cell lines, such as U87 growing in the presence of EGF and fibroblast growth factor (FGF). Simultaneously, the same study showed that GSCs exhibit low levels of phosphorylated mTOR and AKT, noting a connection between the activation statuses of mTOR or AKT with autophagy [198]. Enrichment of GSCs with CD133 marker allows autophagy induction, which results in the expression of isoform II of LC3, ATG5 and ATG12 in comparison with the CD133-negative subtype [155]. In that study, the isolated glioma cells expressed high viability, which correlated with the expression of isoform II of LC3, ATG5 and ATG12, known for their contribution to pro-survival autophagy. The Becn1 gene, which encodes the Beclin 1 protein, was one of the first discovered as autophagy-related proteins, and breakthrough research started to alter with the discovery of the Beclin 1 initiator complex [199]. Beclin 1 is a key component of the autophagy initiation complexes in normal and pathological conditions such as glioblastoma [200,201,202]. Our study suggests that some GSCs, including those isolated from patients, showed a decreased or increased expression of Beclin 1 in comparison with normal astrocytes [203]. Moreover, no correlation between Beclin 1 expression and detection of p62 or the pro-autophagy isoform II of the LC3 proteins was observed, proposing a disbalance between the expressions of autophagy-related proteins in GSCs. Whether all autophagy-related proteins change their expression levels during the development of GSCs remains to be uncovered. For instance, a recent report suggests that TMZ treatments launch a tumor escape mechanism in serum-free conditions through matrix metalloproteinase-14 (MMP14) upregulation and activation of GSC stemness [204]. At the same time, a distinct effect of autophagy induction was observed in the patient-derived glioma cells grown in serum abundance conditions. It occurs that increasing the TMZ concentration was associated with the enhancement of autophagy for the serum-cultured G113 and G116 cells in comparison with populations cultured under serum-free conditions [205]. Collectively, the autophagic mechanism of GSC resistance to therapy represents a big challenge for understating and modulation. The stages of tumor progression in glioblastoma are shown in Figure 4.

## 6. A Tail of Artificial Autophagy Signaling: Neural Stem Cells (NSCs), Astrocytes or Glioblastoma Mutations?

How tumor acquires autophagic signaling and then utilizes it for its benefits has been addressed only partially. Briefly, glioblastoma mainly occurs from neoplastic astrocytes during their transformation from normal counterparts. In turn, normal astrocytes are transdifferentiated from neural stem cells that had been developed in the subventricular zone (SVZ) of the brain. Therefore, it is plausible to expect activation of autophagy in NSCs. In normal physiological conditions, during embryogenesis of neural cells, autophagy clears damaged organelles and destroys pathogens in lysosomes of NSCs. Even though these cells appeared normal and did not undergo any transformations yet, they exhibited some unique features. Thus, during their neural differentiation, an increased expression of the autophagy-related genes, such as Atg7, Becn1, Ambra1 and LC3, has been detected in vivo in the mouse embryonic olfactory bulb (OB) and cultured OB-derived stem/progenitor cells, suggesting autophagy is vital for NSC maintenance [206]. Another report from Wang et al. also confirms the previous finding [207]. Specifically, depletion of the FIP200 gene is involved in the regulation of NSC, which results in a loss of NSCs and impaired neuronal differentiation. These reports echo an earlier made report that in the SVZ, where NSCs reside, inhibition of autophagy and Beclin 1 regulator 1 (AMBRA1) and Beclin1 expression resulted in NSC apoptosis and decreased cell proliferation [208]. 

However, activation of artificial autophagy can also occur during astrocyte maintenance. Thus, during glioblastoma development from astrocytomas, assessment of mutations to constantly activate autophagy signaling remains an elusive option. In these scenarios, initiating mitochondrial or lysosomal defects and dysfunctional proteasomal pathways, which may cause accumulation of damaged organelles and impaired autophagy, seems reasonable [209]. In 2009, Ferguson et al. demonstrated artificial autophagy signaling in a mouse model of Charcot–Marie–Tooth disease and amyotrophic lateral sclerosis (ALS) disorders [210]. As a result of the mutations located upstream in the PI(3,5)P(2) regulatory complex, the factor-induced gene 4 (Fig4) and Vac14 astrocytes accumulated autophagy proteins LC3-II, p62, etc. have been suggested to contribute to the body of disease. It has been recently shown that astrocytes, upon gaining mutations in the glial fibrillary acidic protein (GFAP), developed autophagy [211]. The molecular-level study suggests that activation of p38 MAPK by GFAP accumulation is partially responsible for the downregulation of phosphorylated-mTOR. Later, mutations/deletions in astrocytes carry on the glucosylceramidase beta (GBA) gene, which during neurons and astrocytes degeneration has been shown to produce autophagy. Recently, gaining mutations in tuberous sclerosis complex (TSC) during the development of subependymal giant cell astrocytomas (SEGAs) in the brain led to the constitutive activation of mTORC1 that is involved in the survival of tumor cells [212]. All this data suggests that NSCs and astrocytes, which were mentioned above, can play role in the activation of autophagy in glioblastoma cells and can hinder the development of autophagy signaling upon glioma progression. 

## 7. Conclusions and Future Perspectives

Glioblastomas are considered to be one of the most invasive, heterogeneous and aggressive neoplasms. The short survival is associated with the recurrence, which is almost inevitable in most cases. The self-renewal abilities of glioblastomas are usually attributed to the developed therapeutic resistance in post-surgical microscopic residual tumor cells. Therapeutic resistance in glioblastoma is explained to be a complex process associated with low cellular levels of pro-apoptotic proteins, high levels of anti-apoptotic proteins, enhanced pro-survival signals and genetic instability. During the last decade, autophagy has been extensively studied and its link with cancer, including glioblastoma pathogenesis, is well documented. Autophagy is an evolutionarily conserved pathway that involves lysosomal degradation of damaged organelles and plays a crucial role in cell homeostasis. Mammalian cells under stress conditions, such as nutrient starvation, hypoxia and ROS generation, initiate protective autophagy pathways to maintain modulations in cellular metabolism. The protective autophagy facilitates the degradation and recycling of cell organelles and proteins necessary for the survival. On the contrary, altered autophagy can cause excessive degradation, leading to autophagic cell death. Thus, the piling number of studies indicate autophagy-mediated cell death as a potent strategy for targeting cancer cells. In the earlier studies, glioblastoma cells treated with TMZ have been shown to express a high level of autophagy signaling. This was explained that protective autophagy was initiated against TMZ treatment stress in glioblastoma cells as a survival mechanism. Moreover, inhibiting this TMZ-induced protective autophagy by an inhibitor of autophagy made the glioblastoma sensitive towards TMZ treatment. However, the strategy of targeting autophagy is debatable, since several research groups claim inhibition of protective autophagy to overcome therapeutic resistance, while others claim inducing excessive autophagy can promote cell death functioning synergistically with apoptosis. It was shown that implementing autophagy inducers enhanced the efficacy of the combination treatment of TMZ with radiotherapy. Thus, these studies strongly indicate the potential of autophagy modulation and its clinical therapeutic applications for the treatment of glioblastomas. 

In the light of recent advancements in the field of the molecular and genetic aspects of autophagy, and specifically its role in glioblastoma therapeutic resistance mechanisms, has changed the question of “how to modulate autophagy” to “when to modulate autophagy”. It is known that the level of autophagy can vary in glioblastoma cells, depending upon the tumor microenvironment conditions. Thus, focusing on the level of autophagy activation in tumor tissues, a therapeutic strategy should be designed to efficiently target survival autophagy. The next question arising in the sequence is the role of GSCs and normal astrocytes surrounding the tumor tissues in a glioblastoma’s therapeutic resistance. Although, these developments have provided a window of opportunity to effectively implement autophagy modulation to target glioblastoma proliferation and therapeutic resistance mechanisms and may provide survival benefits to patients in the clinic. Currently, the role of autophagy in therapeutic resistance has been well documented; however, no autophagy inhibitor or inducer has been approved for clinical practice. Thus, future translational studies are warranted to better understand the role of autophagy in GSCs and the tumor microenvironment and its crosstalk with tumor autophagy. The evidence from clinical trials suggest that CQ and hydroxychloroquine (HCQ) have promising efficacy against glioblastomas. Future studies involving combination of these drugs in a more efficient way could turn the tide. 

## Data Availability

Not applicable.
